# Radiation-Induced Myonecrosis: A Case Report of a Cervical Cancer Patient With a History of Systemic Lupus Erythematosus

**DOI:** 10.7759/cureus.55134

**Published:** 2024-02-28

**Authors:** Shigeo Yamada, Yoshiyuki Fukugawa, Takahiro Otsuka, Tetsuo Saito, Natsuo Oya

**Affiliations:** 1 Department of Radiation Oncology, Kumamoto University Hospital, Kumamoto, JPN; 2 Department of Radiation Oncology, Arao Municipal Hospital, Kumamoto, JPN

**Keywords:** radiation-induced myonecrosis, radiation recall phenomenon, complication of treatment, uterine cervical cancer, concurrent chemoradiotherapy

## Abstract

Radiation-induced myonecrosis is a rare but serious complication of radiation therapy. We present a case of a 49-year-old woman with systemic lupus erythematosus who developed radiation-induced myonecrosis after concurrent chemoradiation for cervical cancer. She underwent external-beam radiation therapy, weekly cisplatin chemotherapy (40 mg/m^2^), and intracavitary brachytherapy. One month later, she received one cycle of nedaplatin (80 mg/m^2^) and irinotecan (60 mg/m^2^). Two months after treatment, she experienced pain in the left inguinal region. An MRI revealed a mass in the left obturator externus muscle and right pectineus muscle suggestive of myonecrosis. A biopsy confirmed the diagnosis. She received hyperbaric oxygen therapy, and her symptoms improved. The masses resolved completely.

## Introduction

Radiation-induced myonecrosis is a rare complication that can occur in skeletal muscle within the irradiated field after radiation therapy [1]. Most radiation-induced myonecrosis appears to be mild [2-3], but it can have a significant impact on quality of life depending on the location of the lesion. They frequently mimic recurrent lesions. Since they often resolve with follow-up, proper diagnosis is important to avoid unnecessary treatment. We present a case of radiation-induced myonecrosis after concurrent chemoradiotherapy followed by nedaplatin and irinotecan in a patient with locally advanced cervical cancer.

## Case presentation

A 49-year-old woman with a history of systemic lupus erythematosus (SLE) presented to the gynecologist with complaints of lower abdominal pain and leg edema. CT and MRI showed a cervical mass extending to the right pelvic wall, as well as enlarged right internal iliac and para-aortic lymph nodes, suggesting malignancy. A biopsy was performed on the mass of the cervix, revealing nonkeratinizing squamous cell carcinoma. The patient was diagnosed with cervical cancer (International Federation of Gynecology and Obstetrics Stage IIIC 2r).

The patient had been taking oral prednisolone (5 mg/day) continuously for SLE but had developed steroid-induced central serous chorioretinopathy one month before the start of the treatment for cervical cancer and had discontinued the medication.

The patient was scheduled for concurrent chemotherapy with radiotherapy (CCRT) in combination with weekly cisplatin (40 mg/m^2^) and external-beam radiation therapy followed by intracavitary brachytherapy. The whole pelvic and para-aortic areas were irradiated with 46 Gy in 23 fractions of 3D-CRT. Boost irradiation of 10 Gy in 5 fractions was added for metastatic lymph nodes. High-dose rate intracavitary brachytherapy of 12 Gy in 2 fractions was performed. Grade 2 gastrointestinal toxicity in the common terminology criteria for adverse events occurred during her treatment, but overall she tolerated the treatment well and completed the planned treatment without chemotherapy dose reduction or interruption. On post-treatment evaluation, the primary tumor showed a complete response based on the response evaluation criteria in solid tumors. A residual tumor remained in the right internal iliac lymph node. Nedaplatin (80 mg/m^2^) and irinotecan (60 mg/m^2^) were administered for one cycle, one month after CCRT.

**Figure 1:**
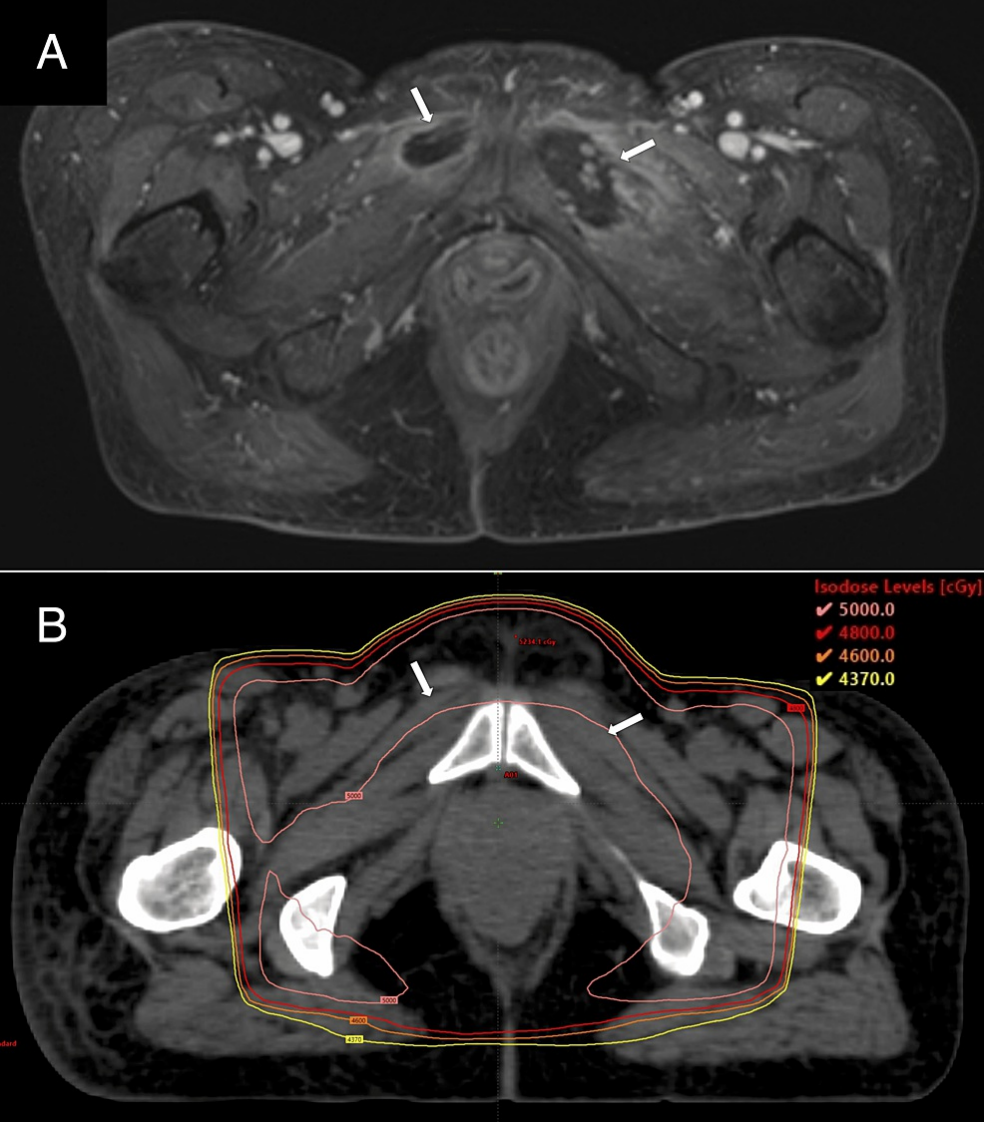
Contrast-enhanced MRI axial image (A) and planning CT axial image showing the dose distribution (B) (A) Contrast-enhanced MRI showed poor contrast mass in the left obturator externus muscle and right pectineus muscle (white arrows). (B) The location of mass was equivalent to 50 Gy in isodose level with a pink line. MRI: magnetic resonance imaging; CT: computed tomography

**Figure 2:**
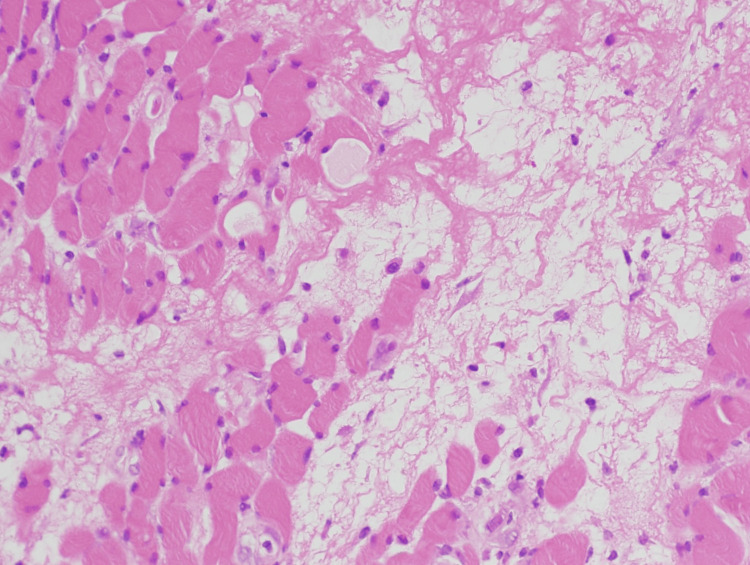
The histopathology of the mass revealed degeneration, atrophy, and necrosis of muscle cells. No malignant cells were seen

Two months after CCRT, the patient complained of left-sided groin pain (numerical rating scale (NRS): eight in 10) and difficulty walking. A physical examination revealed tenderness on both sides of the pubic symphysis but no obvious swelling. Contrast-enhanced MRI showed a relatively well-demarcated mass-like area of poor contrast with marginal enhancement in the left obturator externus muscle and right pectineus muscle (Figure 1A). These areas were involved in the isodose level of approximately 50 Gy (Figure 1B). Since muscle metastasis could not be ruled out, a CT-guided biopsy was performed. The histopathological examination showed strong degeneration, atrophy, and necrosis of muscle cells, but no obvious malignant findings (Figure 2).

**Figure 3:**
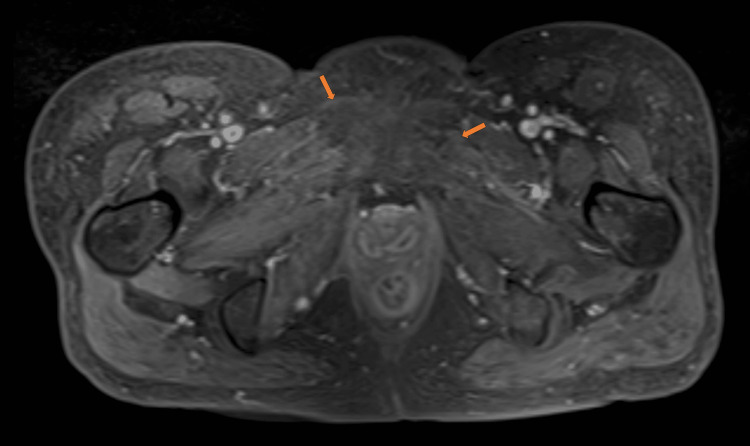
Contrast-enhanced MRI axial image after the first course of hyperbaric oxygen therapy Resolution of the mass in the left obturator externus muscle and right pectineus muscle (orange arrows). MRI: magnetic resonance imaging

The patient was considered to have late-onset radiation-induced myositis because of the necrosis of skeletal muscle within the irradiated field. Since the patient had difficulty walking and a significant decline in quality of life, prompt symptomatic relief was thought to be necessary. Resuming steroid therapy was considered difficult due to the history of central serous chorioretinopathy. As an alternative treatment, hyperbaric oxygen therapy (HBO) was initiated three months after CCRT. Pain control with non-steroidal anti-inflammatory drugs (NSAIDs) and opioids was also performed. After 20 sessions of daily HBO, the pain improved (NRS: 0 in 10), and the patient became able to walk. Four months after CCRT, contrast-enhanced MRI showed the disappearance or obscuration of the mass in the left obturator externus muscle and right pectineus muscle (Figure 3).

**Figure 4:**
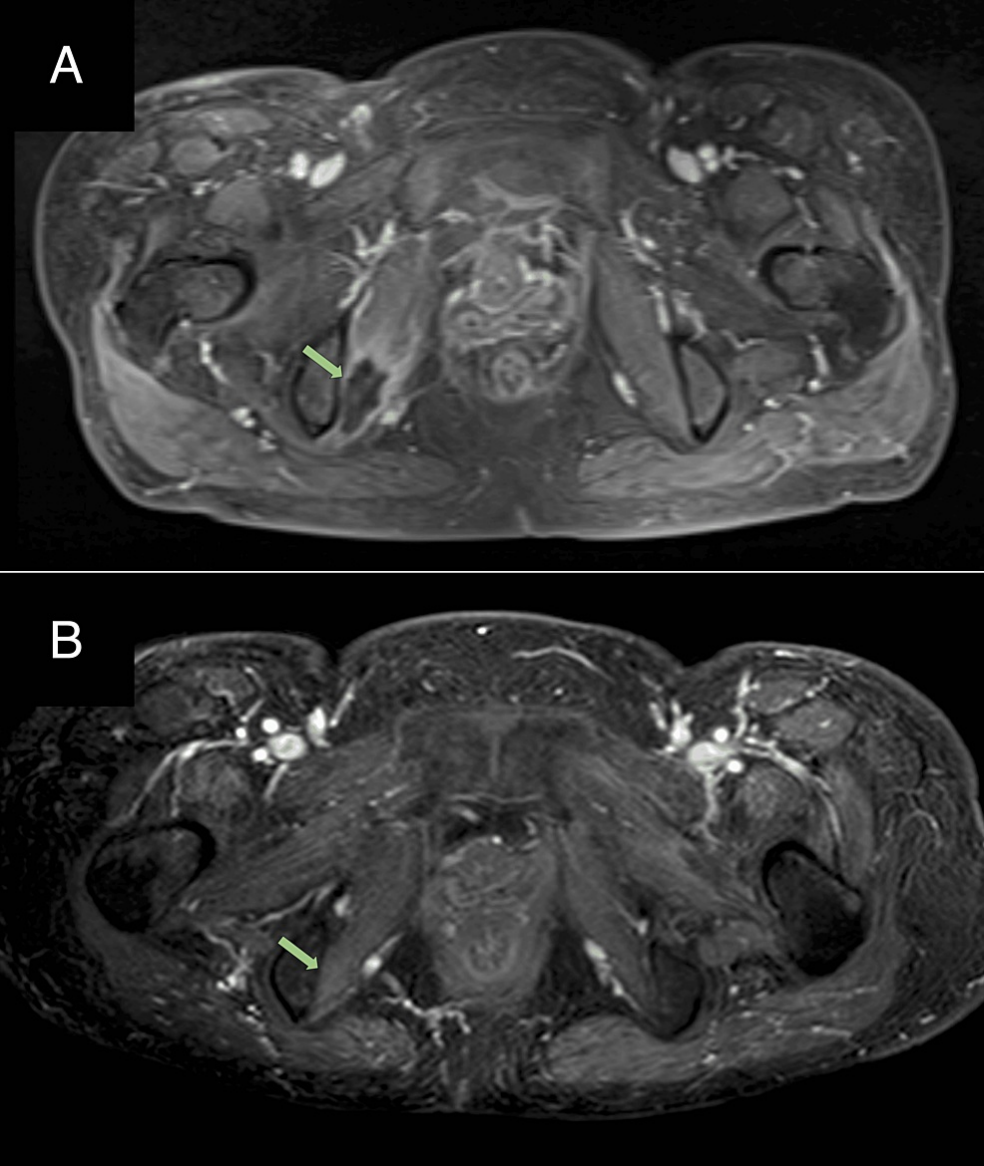
Contrast-enhanced MRI axial image before the second course of hyperbaric oxygen therapy (A) and after the treatment (B) (A) Contrast-enhanced MRI showed poor contrast mass in the right obturator internus muscle (green arrow). (B) The mass was resolved after the second hyperbaric oxygen therapy. MRI: magnetic resonance imaging

However, five months after CCRT, pain in the right inguinal region flared up, and contrast-enhanced MRI showed the appearance of a new poor contrast area in the right obturator internus muscle (Figure 4A), which again corresponded to an isodose level of approximately 50 Gy. A second HBO was performed for the flare-up of myonecrosis, and the pain improved again, and a subsequent MRI confirmed the disappearance of the lesion (Figure 4B).

## Discussion

Myonecrosis is considered to be an infarction of the skeletal muscles that presents clinically as a localized, rapidly enlarging tender mass. Skeletal muscles usually have abundant blood flow, and infarction rarely occurs, but if collateral blood flow is impaired by trauma, vasculitis, or infection, they can develop necrosis [4]. Diabetes, alcoholism, and other etiologies are also often associated [5].

Skeletal muscles are regarded as radio-resistant to therapeutic doses used in conventional radiotherapy, and cases of late radiation-induced myonecrosis have been seldom reported in the literature [1-3,6-7]. We summarize the reported cases in Table 1. On the other hand, Cunningham et al. reported that in a study of myonecrosis in cancer patients, radiotherapy was involved in 34 of the 75 cases detected by MRI [8]. This implies that there might be a considerable number of cases that are unintentionally overlooked, including those without clinical symptom presentation.

**Table 1:** A literature review of cases of radiation-induced myonecrosis M: male; F: female; fr.: fraction(s); ICBT: intracavitary brachytherapy; NSAIDs: non-steroidal anti-inflammatory drugs

Author	Age, sex	Disease	Chemotherapy	Prescribed radiation dose/fractionation	Time interval (radiotherapy to onset)	Myonecrosis treatment	Outcome
Olivotto et al., 1989 [6]	31, F	Cervical cancer	Not administered	40 Gy/20 fr., 50.4 Gy/3 fr. (ICBT)	Three months	Removal of necrotic organs	Patient’s death
Redvanly et al., 1992 [1]	65, M	Hypopharyngeal cancer	Not administered	59.6 Gy/22 fr.	Seven months	Resection	Symptoms improved
Horan et al., 2006 [2]	58, M	Lung cancer	Gemcitabine	24 Gy/8 fr.	13 weeks	Analgesics	Gradually settled
Velcheti et al., 2007 [3]	60, M	Lung cancer	Paclitaxel, carboplatin	70 Gy/35 fr.	Six months	None (follow-up)	Resolution
Brown et al., 2014 [7]	17, M	Metastatic osteosarcoma	Gemcitabine, docetaxel	50 Gy/5 fr.	Two months	Opioid analgesics	Partial resolution
Our data	49, F	Cervical cancer	Cisplatin, nedaplatin, irinotecan	46 Gy/23 fr., 10 Gy/5 fr., 12 Gy/2 fr. (ICBT)	Two months	Hyperbaric oxygen therapy, NSAIDs	Resolution

The physiopathological mechanism of radiation-induced myonecrosis is not clearly understood. Small arteries and capillaries receiving radiotherapy may develop endothelial cell necrosis, intimal proliferation, and collagen deposition. These vascular changes are presumed to cause subsequent inflammation and ischemia and may result in organ necrosis.

Estimating the cause behind the characteristic progression of this case is challenging, given the use of multiple chemotherapy regimens with radiotherapy. The lesions were localized in the bilateral inguinal region, which was the site of irradiation with a dose of approximately 50 Gy. Although equivalent to 110% of the prescribed dose, we assume that it was a common therapeutic intensity for whole pelvis irradiation for cervical cancer and was almost within the tolerable dose for the muscle [9]. Plus, it was noteworthy that the patient presented with an almost total inability to walk due to severe pain after only two months of the first course of adjuvant chemotherapy. Based on a review of similar cases in the literature, the time to onset of myonecrosis in the present case seems to have appeared relatively earlier than in others. Therefore, we hypothesize that the myonecrosis observed in this patient might have been influenced either by a radiation recall phenomenon (RRP) derived from the synergistic effects of radiation and chemotherapy or by an accelerated late radiation toxicity potentially linked to SLE.

The RRP is an inflammatory reaction that occurs when certain systemic agents are administered after radiation therapy. The patient received irinotecan and nedaplatin as additional chemotherapy for residual disease after radiation therapy, and these cytotoxic anticancer agents may have exacerbated late radiation-related adverse events. Dermatitis is a well-known manifestation, but myositis has also been reported occasionally as RRP. Although there are no reports in the literature on radiation-induced myonecrosis as RRP, it is suggested that myonecrosis could occur with the combination use of cytotoxic anticancer drugs as presented in Table 1. Radiation-recall myositis, particularly induced by regimens containing gemcitabine, has been reported relatively frequently. One report also suggests a link with irinotecan [10]; to our knowledge, however, no reports indicate an effect related to nedaplatin. In this case, radiation-recalled myositis induced by either a single agent or both agents might have subsequently developed myonecrosis.

It has long been recognized that patients with collagen vascular disease (CVD) are at increased risk for acute and late radiation toxicity, so SLE may have been an additional risk factor in this patient. However, in recent years, there has been a reevaluation of the relative and absolute contraindications to radiation therapy, and multiple meta-analyses have reported an increased risk of acute and late adverse events in radiation therapy for CVD. Lin et al. reported that increased toxicity above grade 3 is relatively acceptable in patients with CVD and that treatment should not be withheld for patients who require radiation therapy [11]. Shaikh et al. reported that the CVD group was more than twice as likely to develop acute and late grade 2 or higher radiation toxicity compared to the control group, but concluded that it was still an acceptable toxicity [12]. Both meta-analyses have the limitation of including many CVD subtypes and many irradiated sites, and no definite consensus has been developed so far. Also, the correlation between the activity and severity of CVD and its associated toxicity remains unclear. The patient had ceased taking oral corticosteroids for SLE before treatment; however, there was no evident relapse of SLE activity during the radiotherapy period.

Before management for radiation myositis or myonecrosis, it is recommended that the causative agents be withdrawn, provided they are RRP-derived. NSAIDs are an appropriate initial therapy for radiation myositis, but if NSAIDs are ineffective, corticosteroids may be considered. If the patient is refractory to this treatment or, as in the present case, the use of corticosteroids is prudent due to background disease, HBO can be considered an aggressive approach. HBO is supposed to promote the angiogenesis of irradiated tissues and the activation of fibroblasts and is an effective means of treating soft tissue destruction due to a variety of causes, not limited to radiation injury [13]. Feldmeier et al. reported that HBO was an effective treatment for patients with radiation-induced chest wall necrosis [14]. Nevertheless, the literature reporting the efficacy of HBO in soft tissue necrosis of the pelvic region is limited. The present case suggests that HBO may also be effective as a treatment for radiation necrosis of the pelvic region.

## Conclusions

Radiation-induced myonecrosis is a relatively rare adverse effect after pelvic radiotherapy, and if it presents as tumor-like lesions around the primary focus or within the pelvis, it is often difficult to differentiate from tumor recurrence. To avoid unnecessary treatments, close follow-up, careful clinical examination, and, if necessary, a biopsy are required. In the present case, it is difficult to determine the influence of additional chemotherapy and concurrent SLE. However, considering the relatively early onset of myonecrosis and severe symptoms regardless of standard treatment intensity, it may be possible that these factors have been involved in the development of myonecrosis.
